# Drought and High Temperature Stress in Sorghum: Physiological, Genetic, and Molecular Insights and Breeding Approaches

**DOI:** 10.3390/ijms22189826

**Published:** 2021-09-11

**Authors:** V. B. Rajendra Prasad, Mahalingam Govindaraj, Maduraimuthu Djanaguiraman, Ivica Djalovic, Anjali Shailani, Nishtha Rawat, Sneh Lata Singla-Pareek, Ashwani Pareek, P. V. Vara Prasad

**Affiliations:** 1 Department of Crop Physiology, Tamil Nadu Agricultural University, Madras 641 003, India; prasadvenugopal@gmail.com; 2 International Crops Research Institute for the Semi-Arid Tropics (ICRISAT), Patancheru 502 324, India; 3 Institute of Field and Vegetable Crops, National Institute of the Republic of Serbia, Maxim Gorki 30, 21000 Novi Sad, Serbia; maizescience@yahoo.com; 4 Stress Physiology and Molecular Biology Laboratory, School of Life Sciences, Jawaharlal Nehru University, New Delhi 110 067, India; anjalishailani@gmail.com (A.S.); nishtharawat475@gmail.com (N.R.); ashwanip@mail.jnu.ac.in (A.P.); 5 Plant Stress Biology, International Centre for Genetic Engineering and Biotechnology, New Delhi 110 067, India; sneh@icgeb.res.in; 6 National Agri-Food Biotechnology Institute, Mohali 140 306, India; 7 Department of Agronomy, Kansas State University, Manhattan, KS 66506, USA; vara@ksu.edu

**Keywords:** drought, high temperature, mechanisms, genetics, breeding

## Abstract

Sorghum is one of the staple crops for millions of people in Sub-Saharan Africa (SSA) and South Asia (SA). The future climate in these sorghum production regions is likely to have unexpected short or long episodes of drought and/or high temperature (HT), which can cause significant yield losses. Therefore, to achieve food and nutritional security, drought and HT stress tolerance ability in sorghum must be genetically improved. Drought tolerance mechanism, stay green, and grain yield under stress has been widely studied. However, novel traits associated with drought (restricted transpiration and root architecture) need to be explored and utilized in breeding. In sorghum, knowledge on the traits associated with HT tolerance is limited. Heat shock transcription factors, dehydrins, and genes associated with hormones such as auxin, ethylene, and abscisic acid and compatible solutes are involved in drought stress modulation. In contrast, our understanding of HT tolerance at the omic level is limited and needs attention. Breeding programs have exploited limited traits with narrow genetic and genomic resources to develop drought or heat tolerant lines. Reproductive stages of sorghum are relatively more sensitive to stress compared to vegetative stages. Therefore, breeding should incorporate appropriate pre-flowering and post-flowering tolerance in a broad genetic base population and in heterotic hybrid breeding pipelines. Currently, more than 240 QTLs are reported for drought tolerance-associated traits in sorghum prospecting discovery of trait markers. Identifying traits and better understanding of physiological and genetic mechanisms and quantification of genetic variability for these traits may enhance HT tolerance. Drought and HT tolerance can be improved by better understanding mechanisms associated with tolerance and screening large germplasm collections to identify tolerant lines and incorporation of those traits into elite breeding lines. Systems approaches help in identifying the best donors of tolerance to be incorporated in the SSA and SA sorghum breeding programs. Integrated breeding with use of high-throughput precision phenomics and genomics can deliver a range of drought and HT tolerant genotypes that can improve yield and resilience of sorghum under drought and HT stresses.

## 1. Introduction

Sorghum (*Sorghum bicolor* L. Moench) is widely grown as a staple food in arid and semi-arid regions of Sub-Saharan Africa (SSA) and South Asia (SA) for its food and fodder use. Drought and high temperature (HT) stresses are the most important abiotic stresses limiting sorghum yield potential in arid and semi-arid regions, leading to food and nutritional insecurity in SSA and SA. Drought is defined as a prolonged shortage of plant available water, primarily due to insufficient rainfall or precipitation. It can also occur due to exceptionally HTs and low humidity driving the evapotranspiration in plants. Drought is one of the significant environmental factors affecting crop growth and productivity worldwide. Several sorghum producing regions in Africa are vulnerable to drought stress [1]. Occurrences of drought are projected to increase in future climates [2]. However, impacts of drought and its effects on crop production are dependent on rainfall distribution patterns rather than on the total seasonal rainfall. Therefore, drought has become an acute problem for crop growth and development, especially in tropical regions [3]. Drought stress affects almost all the developmental stages of a plant; however, seed germination and early seedling growth phase [4] and reproductive stages are highly sensitive and critical, including in sorghum [5]. Drought stress decreases carbon assimilation, stomatal conductance, and cell turgor, thereby reducing crop normal growth and development and limiting yield [5,6]. Visible moisture stress symptoms on crop plants include wilting of leaves and reduction in leaf area, bud/flower formation, sink numbers and overall growth and yield [5,7]. 

Another important abiotic stress limiting crop production is HT. The temperature beyond the physiological optimum that disturbs the normal growth and development is considered as HT stress. In the future, unexpected short or long episodes of HT stress are predicted to occur more frequently [2,8], which can cause a severe yield decrease. It is projected that the global air temperature may increase by 3.7–4.8 °C by the end of the 21st century [8], due to increases in carbon dioxide concentrations and other greenhouse gases [9]. Studies have shown that frequent HT events and increased mean temperatures will destabilize food systems and threaten local and global food security [8]. In arid and semi-arid regions of SSA and SA, the air temperature is often well above the optimum (32 °C) for sorghum growth and yield. Temperature increases above optimum can decrease yields of sorghum [5] and wheat (*Triticum aestivum* L.) [10], and other food grain crops. Therefore, the expected increases in HT will significantly disturb sorghum production in semi-arid regions of SSA and SA. 

Sorghum breeding programs target two types of products, namely variety (using a population improvement approach) and hybrid (using a heterosis breeding approach). The type of product further depends on the trait inheritance patterns (additive and non-additive), growing environment (homogenous or heterogeneous), and availability of farm inputs including nutrients and irrigation. Improving the drought tolerance in sorghum has been a long-term breeding goal. Breeding for HT tolerance is an emerging area in sorghum because of the changing climate in sorghum growing regions. Therefore, understanding of drought tolerance (stay green and yield under stress) is relatively better understood compared to HT tolerance. However, genetic as well as breeding gaps exist in both stresses in addressing the required tolerance levels in cultivated sorghum varieties or hybrids. 

The above facts indicate that the variability in rainfall patterns and increased air temperature in semi-arid regions are associated with decreased sorghum grain yield. Therefore, it is essential to understand traits and mechanisms that are directly and indirectly affected by drought and HT stress in sorghum. Understanding such trait-associated mechanisms will improve the breeding pipelines to develop a drought or HT sorghum variety or hybrids to sustain the grain yield. This review provides an overview of the impacts of drought and HT stress on sorghum and highlights various physiological, genetic, and molecular insights, key traits, and genomic resources to enhance drought or HT tolerance and their utilization in sorghum breeding programs. 

## 2. Sensitive Stages to Drought or High Temperature Stress

In general, reproductive stages of sorghum development are relatively more sensitive to environmental (abiotic) stresses compared to vegetative stages [5,7,11]. In sorghum, the drought stress-sensitive stages are the panicle development, flowering, and grain filling stages [11]. Similarly, for most cereals, the reproductive stage is more sensitive to HT stress than the vegetative stage, as the former leads to greater yield loss than the latter. In sorghum, the periods between 10 d and 5 d before anthesis (the micro- or mega-sporogenesis stage) and between 5 d before and 5 d after anthesis were most sensitive to HT, causing maximum decreases in floret fertility, leading to a poor seed set [12]. However, like HT stress, the more refined stage sensitivity to drought stress from the vegetative to grain maturity stage needs further attention to reveal the trade-off patterns. 

## 3. Physiological Traits Associated with Drought Stress Tolerance

An overview of various traits associated with drought tolerance in sorghum was summarized by Prasad et al. [5]. In sorghum, stay green is an integrated drought adaptative and a constitutive trait. The ability of the plant to retain chlorophyll molecules during the post-flowering stage, resist lodging, and produce well-filled grain is typically referred to as ‘stay green’ [13,14]. Sorghum line B35, a derivative of a cross between Ethiopian durra and Nigerian landrace, is the most promising line harboring the ‘stay green’ phenotype and widely used as a trait source across regions [15]. In addition, a few promising sources of stay green traits in sorghum were also identified [15,16]. Drought aggravates the chlorophyll loss in susceptible genotypes resulting in less expression of stay green. Delay in the onset of leaf senescence or rate of progression of leaf senescence relates to the functionality of stay green [13]. The leaf senescence rate was negatively correlated with yield under pre- and post-flowering drought stress [14]. The stay green trait is modulated by nitrogen demand by the grain and supply by the leaf through translocation and the roots by uptake. Targeting the quantitative trait loci (QTL) associated with the ‘stay green’ phenotype has identified four QTLs, namely, *Stg1*, *Stg2*, *Stg3*, and *Stg4*, which are collectively responsible for nearly 54% of phenotypic variance observed in stay green sorghum genotypes. Chromosome mapping has tagged the QTLs *Stg1* and *Stg2* on the 3rd chromosome and *Stg3* and *Stg4* on the 2nd and 5th sorghum chromosomes [17]. In addition, it was observed that the stay green trait is associated with the water supply and water loss phenotype [18]. Sorghum genotypes harboring the stay green QTLs reduce the tiller number, canopy size, and coverage and thus reduce the water use during the vegetative stage and make more water available for use during the post-anthesis drought stress, which coincides with the grain filling period [17,19]. However, detailed root architecture of the stay green genotype needs to be explored to understand root-to-shoot communication under drought stress. A recent study indicates that canopy size at pre-flowering is weakly associated with stay green, whereas the canopy size at post-flowering is highly associated with leaf senescence [20]. Thus, the sorghum stay green phenotype contributes to grain yield both under well-watered as well as drought stress conditions.

In plants, one of the essential and prerequisite phenotypes to withstand drought is long and branched roots to penetrate deep and extract the moisture available in the deep layers of soil [21]. Bawazir and Idle [22] reported that the sorghum genotypes having a higher number of seminal roots with large vessel diameters showed more drought tolerance. The genotype with the longest root length with the smallest diameter captured more soil water than the genotype with a shorter root length and large diameter. These results indicate that drought tolerance in sorghum is primarily associated with root length rather than root diameter. Furthermore, root angle is positively correlated with the soil penetration capacity, and a wide root angle reduces the energy demand required for its penetration [23,24]. A narrow root angle with deeper roots allows exploration of soil moisture in deeper layers of stored soil moisture, while a wider root angle with shallow roots allows exploration of soil moisture in the top layers of soils. Studies indicated that deep and better rooting systems improve yields of crops [25,26,27,28,29]. The root system architecture can be manipulated through genetic selection and irrigation management to ensure optimal performance under deficit soil moisture [28,29,30,31].

Osmotic adjustment is defined as a net solute increase leading to lowered tissue osmotic potential. As the water potential is decreased due to lower osmotic potential, water movement from higher concentration (soil) to lower concentration (tissue) occurs, resulting in enhanced turgor [31,32]. Following the relief of stress, the accumulated solutes are decreased [33]. In addition, there is diurnal variation in osmotic potential and net solute accumulation. Sorghum can generally adjust its leaf area, shoot development, canopy architecture, and leaf surface properties according to soil moisture availability [33]. As the minimum leaf water potential decreased at approximately 0.15 MPa per day, the leaves adjusted osmotically at a rate of at least 0.1 MPa per day in sorghum [34]. The stomatal aperture closes at −1.4 MPa to −1.5 MPa of leaf osmotic potential to avoid water loss under severe drought stress. At low leaf potential, around −1.4 MPa, stomata remain closed, which is associated with a rise in the abscisic acid level. If the leaf potential reduces further to −3.7 MPa, swelling of the outer chloroplast’s membrane occurs [34]. Sorghum genotypes exposed to post-anthesis drought stress showed high osmotic adjustment and produced 24% higher yield than their control, which had a low adjustment mechanism. The yield difference was attributed to grain size, and grain number variations are associated with a higher harvest index [35]. Compared to rice (*Oryza sativa* L.) and wheat (*Triticum aestivum* L.), the osmotic adjustment traits are less explored in sorghum. The QTLs and genes related to osmotic adjustment and transgenics in sorghum expressing osmotic adjustment genes have not been studied in detail. Therefore, the osmotic adjustment trait needs further exploration in sorghum.

Canopy temperature, a surrogate trait for stomatal conductance, is directly related. Sorghum can maintain its stomata in open conditions even at low water potential and under a wide range of leaf turgor [36]. In general, plants with high stomatal conductance transpire more and thus maintain a cooler canopy temperature. Therefore, canopy temperature depression (CTD) is a parameter for studying variations across different crop genotypes for transpiration, stomatal conductance, and water use [37]. There were correlations observed between canopy temperatures, WUE, and grain yield in grain sorghum that can be explored for improving drought tolerance [38].

Conservation of water use during early stages of crop development even under high evaporative demand can help with drought tolerance. This trait is termed limited transpiration and is expressed under high vapor pressure deficit (VPD) and was observed in sorghum genotypes [39,40,41]. It can be exploited to enhance drought tolerance in sorghum.

## 4. Physiological Traits Associated with High Temperature Stress Tolerance

Maintenance of membrane stability under HT stress is essential for optimum photosynthesis and respiration. In general, maintaining membrane stability under a stressful environment is an adaptation mechanism because cell membranes are the primary target for abiotic stresses, especially HT stress [42]. Increased cell membrane stability is associated with decreased reactive oxygen species (ROS) production and increased antioxidant levels and antioxidant enzyme activity [42,43]. The major sites of ROS production are chloroplast photosystem (PS) I and PS II reaction centers, peroxisomes, and mitochondria. Decreased membrane stability reflects the lipid peroxidation of the membranes caused by ROS. In addition, the reduced utilization of ATP (adenosine tri phosphate) and NADPH_2_ (nicotinamide adenine dinucleotide phosphate—reduced) in dark reactions (Calvin-Benson cycle), which are produced during light reactions, enhances ROS formation due to stomatal and non-stomatal limitations [44]. Manavalan and Nguyen [45] indicated that chlorophyll fluorescence measurements could be a potential non-destructive tool to measure HT tolerance because the Fo/Fm ratio of chlorophyll a fluorescence can be considered as thylakoid membrane damage [46]. Studies have documented high genetic variability and heritability for this trait. In addition, the unsaturation level of plastidic and extraplastidic glycerolipids of the leaf was deceased under HT stress as an adaptation mechanism [47]. Detailed research on lipids may help in identifying lipid molecular markers for HT stress improvement in sorghum.

The impact of HT on respiration is less understood. With the increase in air temperature, the respiration rate increases and reaches a point where the carbon demand for respiration cannot be compensated by carbon assimilation through photosynthesis, resulting in carbon starvation [48]. The response of respiration to HT depends on the age of the crop. In general, the respiration rate increases exponentially from 0 to 35–40 °C and reaches the peak value 40–45 °C, and a further increase in temperature decreases the respiration rate [49,50]. The increase in respiration rate indicates increased consumption of assimilates for maintenance or growth respiration. High day or nighttime temperatures increased the night respiration rate [51]. Overall, increasing the efficiency of respiration can lead to increases in growth rates, yield, or tolerance to HT stress and to efficient use and partitioning of carbon [5]. A rice genotype that was susceptible to HT had greater respiration rates and had lower yield compared to a tolerant genotype [52]. Diurnal changes in respiration rate, genotypic variability in response to HT stress, and QTLs for respiration-associated traits have been less explored in sorghum and need to be studied in detail.

In sorghum, grain yield is the final product of the number of spikes/panicles per plant, number of grains per spike/panicle, and individual grain weight. The number of seeds (or >80% seed set) is a good indicator of reproductive success, which is dependent on floret fertility (viability of gametes). Selection for improved seed set and seed numbers under HT will be helpful if a reduction in individual seed size does not offset increased seed numbers. High temperature during gametogenesis or anthesis is associated with reduced seed set percentage due to decreased pollen production, pollen, or stigma (gametes) viability and pollen germination tube growth, an unsuccessful fertilization process, and early embryo abortion [42,47,53]. The loss of pollen viability under HT stress is associated with degeneration of the tapetum layer and/or altered carbohydrate metabolism [53]. The tapetal cells are involved in providing the necessary carbohydrate and nutrients for the developing pollen grains; premature degeneration of tapetal cells under HT stress causes sterile pollen under drought [54] and HT stress [55] in wheat. Jain et al. [56] observed a decrease in carbohydrates in the anther wall and pollen of sorghum along with reduced pollen germination under HT. The individual grain weight is a product of the rate and duration of grain filling. Under optimum growing conditions, a reduction in seed number can increase individual seed weight because of increased assimilate availability per grain [51]. However, under HT stress at early flowering, which led to decreased seed, numbers did not result in increased seed size in grain sorghum [12]. Ovary development in sorghum was associated with genotypic differences in grain weight [57]. Singh et al. [58] observed that the effect of temperature on seed-set percentage was much greater than the effect on individual seed weight, indicating that a reduced seed set in temperature susceptible genotypes was not compensated for by increased seed weight. Sorghum genotypes with a higher seed filling rate and longer seed filling duration within the physiological maturity range (days) under HT stress can provide higher grain yields.

A few other potential traits associated with HT stress tolerance such as early morning flowering and canopy temperature depression in sorghum are reported by Prasad et al. [5]. An early morning flowering trait will allow plants to avoid or escape HT that occurs later in the morning. Genotypes of rice varied in their time-of-day of anthesis among species and cultivars [59] and this must be explored in sorghum. The ability of plants to cool their canopies under HT is another trait that has potential to avoid high canopy or tissue temperatures. Mutava et al. [60] identified different sorghum genotypes with a cooler canopy (escape) and high canopy temperature (tolerance) with higher grain yield.

## 5. Mechanisms Associated with Drought Tolerance

In response to drought, plants exhibit various morphological, physiological, and biochemical adaptive mechanisms to cope and survive under adverse conditions. These responses can be broadly classified into drought escape, drought avoidance, and drought tolerance [61]. The genotypes with drought escaping mechanisms complete their lifecycle before the onset of severe drought stress. In most field experiments, an early flowering phenotype is critically considered as a main drought escape selection index over yield under drought situations. Apart from this, germplasm exhibiting differential responses towards photoperiodism and homeostasis for heading date is considered a drought escaping sorghum phenotype [62].

**Figure 1:**
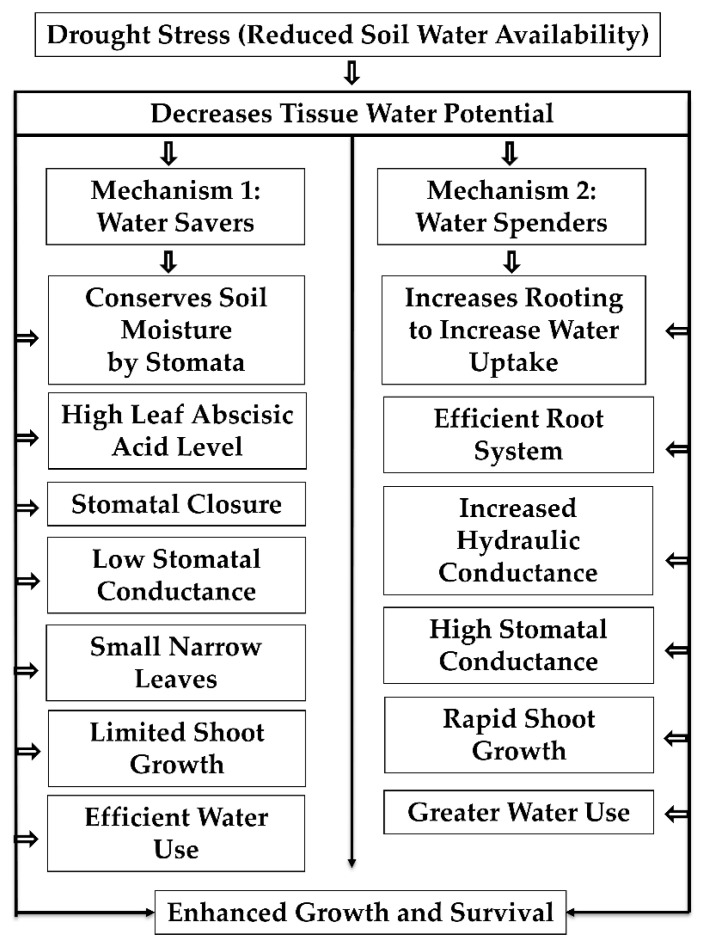
Drought tolerant mechanisms in crop plants. Reduced soil moisture causes drought stress plants to adapt either water saver or water spender mechanisms. The plants expressing water saver mechanisms conserve soil moisture for later stages of growth through reduced transpiration achieved by decreased leaf area, stomatal conductance, and increased abscisic acid levels. In contrast, the water spenders increase rooting depth to improve the water uptake, increase hydraulic conductance, and development of efficient root systems to extract more water from the soil to avoid drought stress.

Crops experiencing drought avoid desiccation by maintaining relatively higher tissue water content either by increased water uptake or by limiting the water loss through transpiration. The crops or genotypes exhibiting the former phenotype are referred to as water spenders and the latter as water savers. In water spenders, the plants avoid the drought by increasing the rooting length, which aids in more water absorption. In contrast, the water savers partially close their stomata, minimize transpiration water loss, and conserve soil moisture. Further, the plant avoids drought stress by activating the osmotic adjustment process, including accumulating compatible solutes [5,63]. Sorghum is known both for its intermittent and terminal drought stress tolerance. Tolerance behavior is attributed to the presence of a dense and prolific root system, ability to maintain a relatively high level of stomatal conductance, and maintenance of internal tissue water potential through osmotic adjustment and phenological plasticity [5,63,64]. The main processes are shown in Figure 1.

## 6. Mechanisms Associated with High Temperature Tolerance

Studies indicate that sorghum yield declines when the temperature crosses the optimum threshold of about 32 °C for grain yield [53,65]. A summary of cardinal temperatures for different growth stages of sorghum has been reported [5]. Thus, in each environment, sorghum’s critical stage must coincide with the optimal air temperatures to realize its potential yield. In general, sorghum adapts and thrives well when the air temperature is between 15 °C and 40 °C. HT-induced decrease in photosynthesis is associated with cell organelles’ structural and functional deformities. The structural deformities include damage to thylakoid membranes, the chloroplast envelope, and chloroplast-protein complexes [47,66,67]. The functional implications of HT on the photosynthesis process include inhibition/or inactivation of the Calvin-Benson cycle enzymes and structural changes in grana.

**Figure 2:**
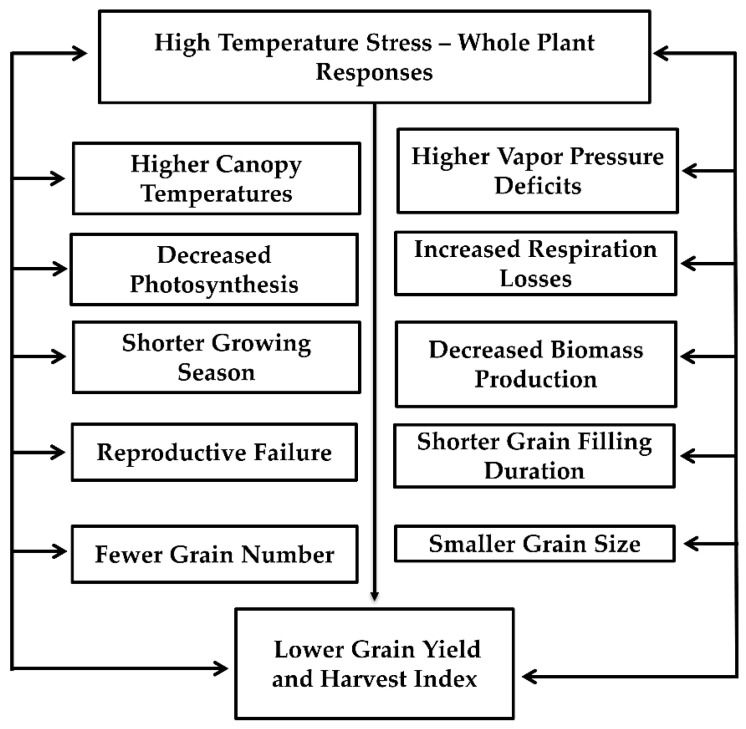
Effects of high temperature stress on physiological and yield processes in grain crops. In brief, when plants are exposed to high air temperatures it leads to higher vapor pressure deficit and increased canopy temperatures, increased respiration, and decreased photosynthesis, leading to decreased biomass production. Higher temperatures shorten the growing season and shorten the grain filling duration, resulting in smaller grain size. Similarly, higher temperature causes reproductive failure leading to lower grain numbers. Fewer grain number and smaller grain size results in lower grain yield and harvest index.

High-temperature stress decreases pigment concentration, photosystem II quantum yield, electron transport system, photosynthesis-related enzyme activities, and gas exchange in plants [66,67]. The enzyme ribulose 1,5-bisphosphate carboxylase/oxygenase (Rubisco) does not appear to limit photosynthesis at HT. However, the activity of Rubisco activase was decreased under HT, resulting in a decreased photosynthetic rate. The effect of HT on chlorophyll and photosynthetic apparatus could be linked with the production of ROS such as superoxide radical (O_2_^−^), hydrogen peroxide (H_2_O_2_), and hydroxyl radical (OH^−^) [67]. High temperatures during flowering and gametogenesis decreased the number of grains per panicle; however, there was no influence on individual grain weight [12]. Membrane thermostability is highly correlated with HT stress tolerance in various crops. At HT, a decrease in the level of unsaturation in both plastidic and extra-plastidic glycerolipids was observed. A summary of HT at whole plant levels is represented in Figure 2.

## 7. Genes Associated with Drought Tolerance

To cope with environmental stress, plants tend to reprogram their gene expression, which subsequently regulates various responses at the molecular, biochemical, and physiological levels. In plants, the role of individual genes that promote and impart stress tolerance has been well known. For example, SbSNAC1 promotes drought tolerance in sorghum [68], but in most cases, a single gene is not sufficient to provide tolerance against a particular or an array of stresses [69]. Instead, transcription factors are master switches, and they orchestrate the expression of several genes that play an essential role in the plant’s adaptation to stresses [70]. Shanker et al. [71] identified a protein in the sorghum genome that has a similarity to the *HARDY* gene in *Arabidopsis* (*AtHRD*). The *AtHRD* contains an APETELA2/ethylene responsive factor (AP2/ERF) domain, which is associated with drought tolerance. Structural prediction indicates that the sorghum homolog of *AtHRD* binds to the GCC box of drought-responsive genes, suggesting its role in drought tolerance in sorghum. The APETALA2 (AP2)/ethylene responsive element binding factor (EREB) subfamily of transcription factors is highly induced in drought tolerant sorghum genotypes (BTx623 [DR1] and SC56 [DR2]) under drought. Apart from AP2/EREB, several abiotic stress-related transcription factors, including bZIP, bHLH, zinc finger, GRAS, and MYB were upregulated in drought tolerant sorghum genotypes [72].

The Dof (DNA binding one finger) transcription factors are known to play a role in drought tolerance in sorghum [73]. The *SbDof* genes, *SbDof12*, *SbDof19*, and *SbDof24,* were upregulated in response to drought, whereas *SbDof6*, *SbDof15*, *SbDof16*, *SbDof18*, and *SbDof20* were downregulated in sorghum under a similar situation [73]. The *SbDof* genes also exhibit time-dependent differential expression in response to drought stress. For example, *SbDof19* and *SbDof24* expressions were high immediately after the onset of drought while *SbDof21*, *SbDof22*, *SbDof23*, *SbDof25*, *SbDof27*, and *SbDof28* were upregulated only at later stages of drought [73]. This differential expression pattern of *SbDofs* supports their role in drought stress tolerance in sorghum. The transcription factor, namely, auxin response factor (ARF), regulates the expression of auxin-responsive genes, including *GRETCHEN HAGEN3* (*GH3*) and *LATERAL ORGAN BOUNDARIES* (*LBD*). The expression of genes *SbGH3* and *SbLBD* was highly upregulated in sorghum under drought conditions, indicating the role of auxin in drought response [73,74]. One of the largest families of plant-specific transcription factors includes NAC regulating several other genes’ transcription in response to abiotic stress. The five members of *SbNAC* (*SbNAC014*, *SbNAC034*, *SbNAC035*, *SbNAC037*, and *SbNAC041*) were found to regulate the response of sorghum under post-flowering drought stress positively. In contrast, *SbNAC052*, *SbNAC073*, and *SbNAC116* were downregulated during the post-flowering drought in sorghum [75]. Moreover, in sorghum genes coding transcription factors, including NAC, HSF, and ethylene-responsive transcription factor (ERF), were upregulated in the leaves, while in roots, the expression of transcription factor coding genes, including *NAC*, *HSF*, *WRKY*, *ERF*, *HD-ZIP*, *MYB*, and *bHLH*, were below optimum even under mild and severe drought stress [76].

*WRKY* family transcription factors have been shown to play a key role during plant adaptation against drought stress. Studies have identified a novel *SbWRKY* gene, namely, *SbWRKY30*, which is mainly expressed in the taproot and leaves of sorghum under drought situations. *SbWRKY30* specifically binds to the W-box element in the promoter of drought stress-responsive gene *SbRD19* in sorghum. The study supports the notion that *SbWRKY30* is a positive regulator in response to drought and is a candidate gene for imparting drought tolerance in several crop plants [77]. Furthermore, other *SbWRKY* genes, *SbWRKY45*, *SbWRKY79*, *SbWRKY83*, and *SbWRKY16*, were highly expressed in sorghum under drought stress [78]. Altogether, the expression patterns of *SbWRKYs* in sorghum under drought suggest their significant role in drought tolerance.

In sorghum, the expression of auxin transporters *SbPIN/5/8/9/11* was highly increased under ABA and drought treatments, whereas *SbPIN/3/6/7/10* were found to be downregulated under similar conditions [79]. Aglawe et al. [80] discovered that auxin response factors (ARFs) also get activated upon drought stress. Fifty differentially expressed sorghum-specific drought-responsive gene orthologs were identified in maize, rice, or *Arabidopsis*, which were previously thought to be non-functional. These orthologs include transcription factors coding ABREs and CGTCA-motifs, or motifs that are involved in the ABA signaling pathway [81].

Glycine betaine is a compatible solute known to be involved in drought tolerance in many plants, including sorghum. Betaine aldehyde dehydrogenase *BADH1/15* expression was found to be induced under drought conditions in sorghum, and its expression coincides with the accumulation of glycine betaine. There was a 26-fold increment in glycine betaine and a 108-fold increment in proline content under water-deficient situations in sorghum [82]. The mannitol-1-phosphate dehydrogenase (*mtlD*) gene from *E. coli* was introduced into sorghum, and the transgenics were found to possess higher leaf water content and better growth than control sorghum plants under drought conditions [83]. *SbP5CS1* and *SbP5CS2* genes play an important part in proline biosynthesis and were found to be upregulated under drought stress [84]. These studies provide evidence for the role of various genes in modulating drought stress tolerance in sorghum.

## 8. Use of Drought Responsive Genes in Developing Tolerant Genotypes

Abou-Elwafa and Shehzad [85] studied genetic diversity among 96 sorghum accessions in association with molecular markers linked with morphological traits under drought stress and assessed the expression profile of drought-responsive genes. They identified three drought-responsive QTLs, i.e., *Xtxp69* on chromosome 3, *SbAGA01* on chromosome 8, and SbAGB03 on chromosome 9, which are associated with the drought-resistant linked phenotypic trait. Furthermore, they also found the upregulated expression of drought-responsive genes using in silico methods, i.e., aldehyde oxidase 3 (*SbAO3*), aspartic protease G 1 (*SbASPG1*), CBL-interacting protein kinase 15 (*SbCIPK15*), cytokinin dehydrogenase 4 (*SbCKX4*), glutathione S-transferase (*SbGST*), G-type lectin S-receptor-like serine/threonine-protein kinase (*SbGsSRK*), mitogen-activated protein kinase 7 (*SbMAPKKK7*), mitogen-activated protein kinase 10 (*SbMAPK10*), pentatricopeptide repeat-containing protein (*SbPPR3*), S-type anion channel (*SbSLAH2*), thaumatin-like protein 1b (*SbTLP1b*), U-box domain-containing protein 43 (*SbPUB43*), WRKY transcription factor 46 (*SbWRKY46*), and zinc finger protein (*SbZFP*). The CKX4 protein family catalyzes the activity of cytokinin dehydrogenase enzymes, which results in either improved root biomass or altered root morphology. Overexpression of *CKX4* has significantly enhanced drought tolerance and influences plant morphology and fertility [86]. *PUB43* is a U-box protein induced by *PUB* under drought stress conditions [87]. Glutathione S-transferases (GSTs) are ubiquitous enzymes and catalyze diverse functions in plants and are found to be induced under both oxidative and drought stress situations in plants [88]. *WRKY46* transcription factor was found to be involved in osmotic and drought stress in *Arabidopsis*. *WRKY* overexpression improved osmotic stress tolerance either by downregulating the expression of its downstream targets or through the regulated expression of the abiotic stress-responsive genes in an ABA-independent manner [89]. Calcineurin interacting protein kinase (CIPK) is a group of positive drought regulators that play a crucial role in plant response towards abiotic stress tolerance, and their expression is found to be upregulated under drought. Furthermore, CIPKs enhance the expressions of several genes associated with drought [90].

Zinc finger protein (ZFP) transcription factor shows elevated expression under drought, and it plays a negative role in drought stress signaling by downregulating the expression of several H_2_O_2_ homeostasis genes [91]. Two mitogen-activated protein kinase genes (*MAPK10* and *MPKKK7*) are involved in the drought tolerance mechanism by regulating stomatal responses via the ABA signaling mechanism [92]. S-type anion channel (*SLAH2*) plays a crucial role in drought stress response by impairing light-induced stomatal opening [93]. Fracasso et al. [94] observed that differentially expressed genes (DEGs) complemented in “response to abiotic stimulus and stress” were upregulated more in a drought tolerant genotype of sorghum. Furthermore, Zhang et al. [95] studied several DEGs responding to mild and severe drought enriched in “response to stimulus”, “water deprivation”, “abscisic acid stimulus”, “temperature stimulus”, and “reactive oxygen species”. In response to mild and severe drought, 66 DEGs were identified, and they belong to *ERF* (six upregulated and three downregulated), *HSF* (six genes upregulated), *bHLH*, *HD-ZIP*, *MYB*, *NAC*, and *WRKY* group of transcription factor families. They showed that several HSP genes responsive to drought stress are pinpointed in a tandem zone of the sorghum genome, which indicated the importance of the HSF transcriptional regulatory system in drought tolerance. Eight chaperone and eleven LEA genes in roots and one chaperone and three LEA genes in leaves were found to be upregulated under mild and severe drought stresses [95].

Li et al. [96] overexpressed the sorghum leucine-rich repeat-receptor-like kinase gene (*SbER2-1*) under maize ubiquitin promoter to improve the drought tolerance in maize by exploiting the *Agrobacterium*-mediated genetic transformation and observed a gradual increase in the expression of *SbER2* and *SbER1* with the severity of drought stress. The transgenic sorghum lines showed delayed leaf senescence, higher photosynthetic rate, and higher lignin content compared to wild type (WT) under drought conditions [96]. Similarly, the sorghum *DREB2* gene was exploited to enhance drought tolerance and yield in rice, and it is also observed that under drought, *rd29A*: *SbDREB2* expressing transgenic rice plants produced more panicles as compared to that of rice lines expressing *SbDREB2* under *CaMV35S* promoter. In *Arabidopsis*, overexpression of *SbSNAC1* confers drought tolerance [90]. Mannitol-1-phosphate dehydrogenase (*mtlD*) gene expressing transgenic sorghum lines exhibit high root growth, more soluble sugar content, and lower malondialdehyde content under drought conditions and thus showed enhanced tolerance under drought conditions [97].

## 9. Genes Associated with High Temperature Stress Tolerance

Proveniers and Van Zanten [98] discovered the role of a basic helix-loop-helix (*bHLH*) transcription factor, phytochrome interacting factor 4 (PIF4) under HT stress, and its orthologs were found in several crops including sorghum (https://www.arabidopsis.org/servlets/TairObject?accession=Locus:2053733, accessed on 21 August 2020). The ARF genes were also induced by HT stress in sorghum; for example, expression levels of nine *SbARF* genes (*SbARF4*, *SbARF7*, *SbARF9*, *SbARF15*, *SbARF17*, *SbARF19*, *SbARF21*, *SbARF22*, and *SbARF24*) were constitutively upregulated after HT stress, while eleven *SbARF* genes (*SbARF1*, *SbARF5*, *SbARF6*, *SbARF8*, *SbARF10*, *SbARF11*, *SbARF12*, *SbARF16*, *SbARF18*, *SbARF20*, and *SbARF25*) induced their expression levels during HT stress [99].

## 10. Genes Associated in Both Drought and High Temperature Stress Tolerance

Drought and HT stress signaling induces the expression of several trans-acting factors, which trigger the expression of drought and HT-responsive genes to help the plants survive under adverse conditions. Mitogen-activated protein kinase (*MAPK*/*MPKs*), Ca-dependent protein kinases (*CDPKs*), sugar (as signaling molecule), nitric oxide (NO), and phytohormone are the main signaling molecules that regulate the expression of several stress-responsive genes [100]. *Drought-hypersensitive mutant1* (*DSM1*) is the drought inducible gene that confers tolerance against drought via the MAPK pathway. Activated stress-responsive genes detoxify the reactive oxygen species (ROS), reactivate the vital structural proteins and enzymes, and maintain the cellular homeostasis, which ultimately leads to tolerance against abiotic stresses, including HT and drought stress [101].

Heat shock transcription factors (HSFs) regulate the expression of heat shock proteins (HSPs), which play a significant role in HT stress response. In sorghum, the expression of different HSFs was induced by HT and drought stress. For example, high-level expression of *HSF1* was observed during HT stress, while *HSF5*, *HSF6*, *HSF10*, *HSF13*, *HSF19*, *HSF23*, and *HSF25* were upregulated during drought [102]. A study on five genotypes of sorghum revealed the role of heat shock transcription factors under drought stress [103]. Heat shock factors *SbHSF5* and *SbHSF13* were upregulated in the leaf tissues of all five sorghum genotypes under drought.

Glutathione reductases (GRs) are known to play an important part in antioxidant machinery. Additionally, rice heterotrimeric G-protein complexes (G α subunit) show high homology with sorghum. Rho-type GTPase-activating protein 1 (RGA1) of sorghum comprises *cis*-regulatory elements such as ABA, ARE, GT-1 boxes, LTR, and MeJAE, suggesting their role in abiotic stress signaling. Further, under drought situations, transcript profiling of *RGA1* showed its upregulation while its expression was downregulated under HT stress. These findings confirm the vital role of G- protein complexes in drought and HT stress tolerance in sorghum. A list of genes along with its function is provided in the Table 1.

## 11. Molecular Marker Resources and Quantitative Trait Loci (QTL) Mapping

Major agricultural traits, including yield, biotic, and abiotic stress responses, are controlled by multiple quantitative trait loci (QTLs). To date, several QTLs have been identified in sorghum that are involved in drought and HT tolerance. Many genetic linkage maps have been constructed in sorghum using molecular markers, such as amplified fragment length polymorphism (AFLPs), restriction fragment length polymorphism (RFLPs), diversity arrays technology (DArTs), randomly amplified polymorphism DNA (RAPDs), and simple sequence repeats (SSRs) [104,105,106,107,108,109,110,111]. These genetic linkage maps lead to the identification of several QTLs for stress tolerance, including drought and HT tolerance.

In sorghum, the drought response depends on the time of stress occurrence, and pre-flowering drought tolerance is most widely studied than post-flowering drought tolerance [106,112,113]. Yet, very limited information is available on genetic mapping and QTLs associated with pre-flowering drought tolerance traits. For the identification of QTLs associated with pre-flowering drought tolerance in sorghum, recombinant inbred lines (RILs) were obtained by crossing two contrasting sorghum genotypes, viz., “Tx7078” (pre-flowering tolerant, post-flowering susceptible) and “B35” (pre-flowering susceptible, post-flowering tolerant). The RILs were genotyped with RFLP and RAPD markers and six QTLs on linkage groups D, F, and M specific for pre-flowering drought tolerance were identified [113]. The expressions of these QTLs were found to be in line with the severity of the drought stress levels. Kebede et al. [112] identified four QTLs on linkage groups C, E, F, and G for pre-flowering drought tolerance from RILs obtained from the cross “SC56 × Tx7000”. A major QTL was found to be on linkage group G, (*Pfr G*) and the locus encompasses an allele linked with a pre-flowering drought tolerance trait introgressed from the tolerant parent TX7000. *Pfr G* contributed 15–37.7% of the phenotypic variations in two different environments, suggesting strong genotype (G) × environment (E) interaction at this locus. *Pfr G* co-localized with QTLs for agronomic traits, including flowering time, stay green, plant height, and lodging resistance, and these three QTLs accounted for 34% variance in the stay green trait [114]. Nineteen QTLs were identified from TX7078 and B35 background associated with drought stress, of which two QTLs accounted for stay green and yield-related traits. Furthermore, three QTLs were identified for the transpiration ratio associated with pre-flowering drought tolerance using 70 RILs of sorghum. These QTLs were found in SBI-09 and SBI-10 and accounted for 17–21% of the phenotypic variance [115]. The QTLs influencing the transpiration ratio co-segregated with agricultural traits, which indicates that the introgression of these QTLs through MAS could help the sorghum crops survive if they experience pre-flowering drought. The post-flowering drought response in sorghum is related to the stay green trait [116]. The stay green trait plays a beneficial role in yield improvement and provides tolerance against drought and HT stresses [117]. Many QTLs for post-flowering drought tolerance (stay green) have been mapped among several RILs in sorghum. Kebede et al. [112] identified three stay green QTLs on linkage groups A, G, and J (namely *Stg A*, *Stg G*, and *Stg J*) from RILs derived from the cross of two contrasting genotypes “SC56 × Tx7000”. Most of the studies on mapping the QTLs for post-flowering drought tolerance used B35 or its derivatives as stay green sources. Sixty-one QTLs for the stay green trait were identified from RILs, derived from a M35-1 (more senescent) × B35 (less senescent) cross, and each trait is controlled by 1–10 QTLs [118]. Each QTL accounted for 3.8–18.7% phenotypic variation. Among these 61 QTLs, *Stg2*, *Stg3*, and *StgB* were expressed prominently under all situations. Borrell et al. [17] showed that the four *Stg* QTLs modulate the sorghum canopy size by reducing the number of tillers, enhancing the size of lower leaves, and decreasing the size of upper leaves and the number of leaves per culm. Mapping of the stay green QTLs on chromosome10 in sorghum from an introgression line cross, RSG04008-6 (stay green) × J2614-11 (moderately senescent), revealed several genes associated with delayed senescence [119].

## 12. Breeding for Drought and High Temperature Stress Tolerance

Direct and indirect traits-based selective breeding are the two basic approaches that are widely followed to screen and develop genetic materials for drought tolerance. Direct selection for drought tolerance is conducted under conditions where stress factors occur uniformly and predictably, whereas indirect selection involves selecting genotypes under managed stress environments closer to or away from target locations. However, environmental factors such as temperature and moisture are highly variable from one location to another and difficult to predict. Variation for stress tolerance exhibits a large environmental component or substantial genotype-by-environment interaction forcing direct selection for a physiological trait under a single environment. As a result, indirect selection breeding is the most preferred selection method based on developmental traits or assessing plant water status and plant function [120].

The objectives in the sorghum drought tolerant breeding program involve identifying a cultivar that produces maximum grain from a given quantity of water (i.e., high water use efficiency). Substantial genetic variations exist among sorghum genotypes for grain yield and water use efficiency. Therefore, the sorghum breeders should find a hybrid that can efficiently utilize available water resources from its surroundings. Thus, understanding root variations among sorghum genotypes should be well investigated and should be included as a major component in the breeding programs aiming towards developing hybrids to cater to the global need. From a crop production perspective, drought tolerance is defined as the crop yield’s consistency under a specific target drought stress environment (broadly the target population environment) due to underlying drought tolerance mechanisms [32,33,36]. However, to be agronomically useful, a drought tolerant cultivar should also have a good yield potential under favorable conditions since it is challenging to predict spatial and temporal drought effects. Concerted breeding towards developing tolerant lines against a specific pattern of drought occurrence in a target region is the best way to improve grain yield under moisture-limited conditions. This task demands the availability of large-scale, cost-effective screening techniques, which can facilitate efficient discrimination of genotypes for drought tolerance. Examining the germplasm for drought adaptive traits is the preliminary and most important step towards identifying drought tolerant genotypes. Sorghum tolerant genotypes [121,122,123] to drought stress at various growth stages are presented in Table 2. These will be useful for further genomics and physiological studies. India has a more than 60% monsoon-based cropping system, and the most effective way to minimize the drought-induced yield losses is through the development of early-maturing genotypes that can escape from terminal or late-season drought. Breeding for early-maturing varieties may not always be associated with higher yield in regions with erratic rainfall patterns. The replacement of traditional, long-duration (130 to 180 days), and open-pollinated varieties (OPVs) with early hybrids and OPVs that mature at 100 to 110 days before the monsoon end or before the onset of late-season drought has resulted in a remarkable increase in rainfed sorghum production. In India, the long duration (>110 days) sorghum varieties experiencing terminal drought, especially during the monsoon season, show a drastic reduction in the yield as compared to that of early-maturing improved sorghum cultivars (100 days) (Table 2).

**Figure 3:**
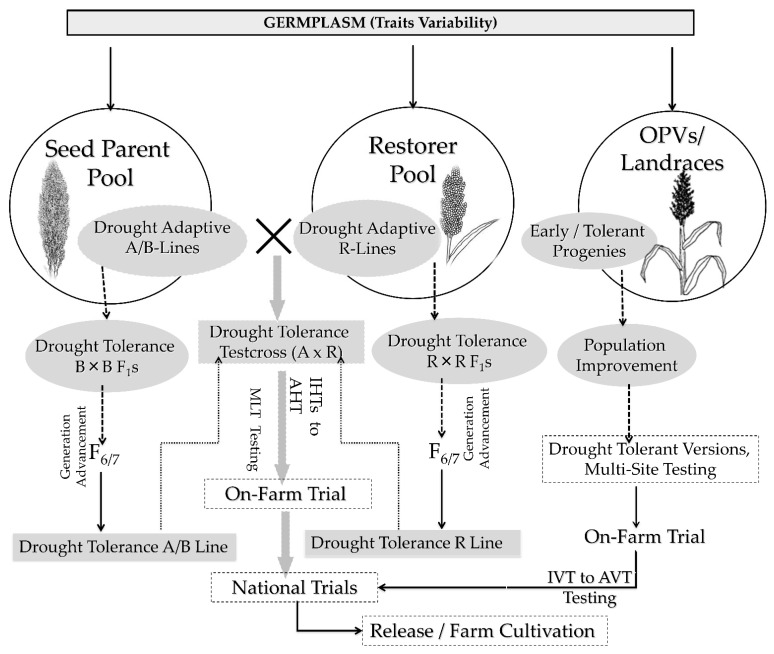
Fast-track breeding approaches for developing variety and hybrids for drought tolerance in sorghum. Briefly, genetic variation for drought tolerance in sorghum is sourced from elite breeding populations or germplasm. In sorghum, development of hybrid requires a male sterile line (A-line), a maintainer line (B-line), and a restorer line (R-line). A-line is male sterile and used as female; B-line is an isogenetic line of A-line and serves as maintainer line, and R-line is a male fertile line used as pollen parent. Identified tolerant lines will be crossed within gene pool (B-lines/R-lines) of sorghum (without interrupt maintainer or restoration genes) to produce recombinants to select and fix in subsequent generations (i.e., F2–F7). At ≥F7 stage, crosses between two gene pool rewards the hybrids population to test in target sites to release a candidate hybrid with tolerance and on parallel, respective B-lines to be converted to its male sterile version (A-line) for commercial production. Various test crosses between A and R lines go through testing at initial hybrid trials (IHTs) to advanced hybrid trials (AHTs) in on-farm tests, whereas in open pollinated varieties (OPVs) breeding, the selected intra-population progenies crosses were recommended to be released as a final product after sequential testing at multiple locations under initial variety trials (IVT) and advanced variety trials (AVT).

Evolving sorghum varieties harboring diverse productivity traits can also perform better towards drought tolerance. For instance, the presence of epicuticle wax on leaf and stalk may reduce evapotranspiration in sorghum under field conditions [124]. The “Physiological Breeding” concept is gaining momentum in the sorghum drought tolerant breeding program, useful for post-rainy breeding pipelines in India. The most critical drought tolerance trait in sorghum is the stay green phenotype [13,14,17]. Several stay green trait introgressed sorghum hybrids has been evolved and are successfully being cultivated across the globe. Borrell et al. [14] reported no decline in sorghum grain yield when stay green sorghum fields were irrigated, but under drought conditions, hybrids with the stay green phenotype out-performed the non-stay green hybrids. Using indirect selection for stay green trait that may have been overlooked in the past but can help improve the drought adaptations in different flowering and maturity groups should be considered in future. Stay green in sorghum also helps improve forage quality besides its water-use efficiency (WUE) and resistance to Anthracnose. Both public and private sector breeding programs uses a similar set of female pool for different end-use products. Strengthening the female parental pipeline with stay green could be more rewarding across end-use products of grain, forage, and dual purposes. To achieve a higher rate of genetic gain in stay green and drought tolerance traits requires a breeding strategy with other key agronomic traits while maintaining focus on sterility, grain quality, and heterosis. The proposed fast-track varietal and hybrid breeding approaches for drought tolerance in sorghum are depicted in Figure 3.

The conventional breeding approaches strongly advocate testing materials in target locations and a selection strategy based on trait genetics. Varieties mostly exploit the highly heritable traits and additive genetic variances to maintain a broader genetic base for varietal plasticity towards different drought situations. Selection and inbreeding are largely exercised to fix the additive genes for a given trait. In contrast, the hybrids express the dominantly inherited traits (non-additive variances) for drought tolerance like other traits. Therefore, one of the two parents (of a hybrid) is adequate to express such dominant traits in hybrids. In this context, examining the inheritance of traits contributing to drought tolerance is essential. The current approach is needed to choose the drought tolerance traits at the screening level and select parents for hybrid and varietal development.

Traditional breeding methods, including pure line selection methods, are exercised in many national and regional sorghum research programs in Africa and Asia. Alternatively, pedigree and bulk selection methods are commonly used in international breeding programs. If the objective is to introgress only a few selected traits relating to drought resistance into a high-yielding cultivar, backcross is the most appropriate breeding method [35]. Drought tolerance breeding pipelines are primarily targeted for the post-rainy season in India and for the rainy season in Africa. For instance, the average yield in the post-rainy is lower (~750 kg ha^−1^) than the rainy season crop in India [125]. In general, genetic enhancement for drought tolerance is accomplished mostly through yield gain under drought. However, selection for yield itself is challenging because of the low heritability of yield. Furthermore, yield under drought is determined by the spatial and temporal variation in the field environment alongside genetic potentials. Therefore, conventional drought tolerance breeding approaches are very slow in progress [126]. Developing genomic tools and the use of diagnostic markers for drought tolerance-associated traits selection in the segregating generations (F2s) will fast track the breeding program. About 40–50% of India’s sorghum production is under post-rainy cultivation prospects for drought resilience varieties or hybrids. The availability of high throughput drought screening techniques, wide genetic variation for traits of relevance, and genomic discovery under different types and magnitude of drought stress are prerequisites for success in selecting desirable genotypes and designing the best-fit product through a multidisciplinary breeding approach in the middle of the 21st century.

Breeding for HT tolerance in sorghum is emerging and, so far, not well described, as this is a complex trait contributed by environments and interaction of various component traits, which are poorly understood from seedling to maturity stage. On the side, the confounding effect of HT and drought is imposing challenges for breeders and physiologists in understanding the HT stress tolerance underlying mechanism in sorghum. Opting for the right breeding method for HT stress tolerance largely depends on the trait genetics discoveries that come from robust screening and phenotyping methods. Identifying the of primary traits for HT is crucial in sorghum followed by measuring the genetic variability for HT among the different sets of genotypes. Variability for HT at seedling stage was demonstrated in sorghum [127]. Understanding the genetic control of HT is a prerequisite for formulating an appropriate sorghum breeding method. To date, positive correlation between grain yield and HT has been used as a viable initial selection criterion for HT. For instance, most tolerant genotypes showed a marginal decrease in seed set at 38 °C, whereas there was a significant reduction reported in susceptible genotypes at 36 °C [52]. The seed set was positively correlated with pollen viability. The seed set under HT stress in field study was correlated with controlled environmental conditions [52]. In sorghum, the most efficient way to minimize the harmful effect of HT is to adjust the planting date so that the critical reproductive stages do not coincide with HT stress. For instance, in a hybrid plot, 50% of its population commences its flowering phase after 65 days, which indicates that the critical growth stage of sorghum will begin approximately 50–60 days after emergence. Therefore, adjusting the sorghum planting date can help to avoid stress and minimize yield loss.

**Figure 4:**
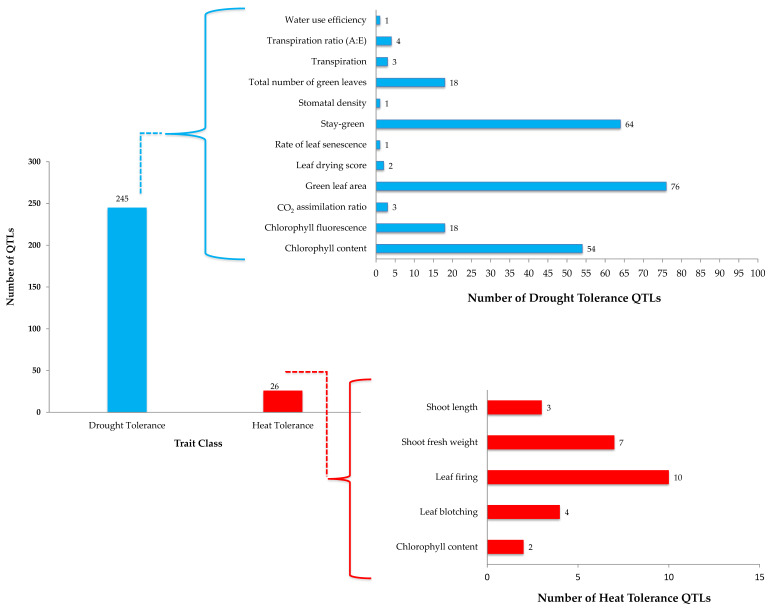
Quantitative traits and Quantitative Trait Loci (QTLs) identified for drought or heat stress in sorghum. Briefly, there were several QTLs reported for abiotic stress tolerance in sorghum. These were grouped into two categories, i.e., drought tolerance and heat tolerance. There were twelve traits associated with drought tolerance with 245 QTLs, while there were 26 QTLs for five traits associated with heat tolerance (HT) in sorghum. Validation of these QTLs and genes under these loci will add value for precise selection and direct transfer major effect QTLs for target traits (drought or HT) in sorghum crop improvement. Represented stay- green, green leaf area and chlorophyl content for drought are widely studied and may help in decoding the probable mechanisms governing drought tolerance genetic control, whereas QTLs for HT are limited and yet to be exploited for potential traits.

Integrated breeding for drought tolerance will be a game changer in future sorghum breeding since more than 240 QTLs have been reported for drought tolerance traits across the globe (Table 3; Figure 4). Of these, almost all of them were physiological and growth factor traits including stay green. These traits are not routinely used in sorghum breeding programs due to various reasons, including (i) availability of screening facility, (ii) availability of low-cost trait markers; (iii) most of these QTLs being on leaf-based assessment and becomes laborious when dealing with a large set of progenies and the breeding traits largely being panicle traits-oriented. The availability of many reported QTLs (Figure 4) opens the avenue for identifying SNP markers–traits association and further validation in the breeding population to select the segregating generation derived from the breeding strategy (Figure 3) for drought-based traits. Leveraging the validated QTLs for drought and use of high throughput phenomics platforms will accelerate the breeding for drought tolerance pipelines.

**Figure 5:**
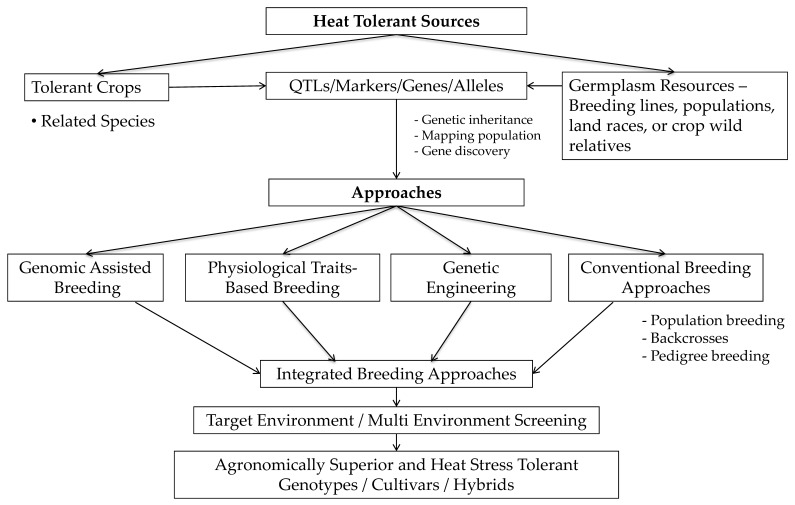
Proposed breeding approaches for development of sorghum heat (high) temperature (HT) stress tolerant cultivars (adopted from Govindaraj et al. [131]). Briefly, HT breeding requires HT sources from the breeding lines or germplasm collections from the source; dissecting HT trait genetics and inheritance in the future will guide the discovery of the candidate genes and loci (QTLs) in future. Identified sources/QTLs/genes can be transferred to elite cultivars that require HT for wider cultivation. To date, no straightforward breeding approach is available for HT. The proposed model is a combination of pre-breeding to product development. Breeding approaches will depend on the mode of trait inheritance (few/major genes or many/minor genes) in conventional breeding or physiological traits-based breeding methods. Genomic approaches will assists the breeding HT sorghum through advanced markers association studies, diagnostic markers, whereas, genetic engineering method helps in gene editing, provided a key candidate gene is identified for HT in sorghum. The reported 26 QTLs for HT in sorghum can be validated and used in regular breeding with support of diagnostic markers (for screening), and forward through genomic selection in breeding pipelines is recommended. Integrated breeding approaches with appropriate testing and screening in target and multi-environments are needed to identify and develop agronomically superior heat tolerant cultivars or hybrids.

The International Crops Research Institutes for the Semi-Arid Tropics (ICRISAT) established leasyscan—a high throughput and precision phenomics platform that can help in dissecting HT tolerance traits in sorghum at seedling stage to initial vegetative stage (30–40 days). This facility may provide new opportunity to capture 3D images and IR images on canopy temperatures and other HT tolerance traits (shoot length, shoot fresh weight, chlorophyll content, leaf firing, leaf blotching). The variability of these traits can improve the understanding of HT tolerance genetic control in sorghum for formulating an appropriate breeding method. Very few sorghum lines (RTx430, BTx3197, RTx7000, and B35) have been studied for HT stress. A study involving these lines and their crosses indicated inbreds were more HT tolerant compared to their crosses for flowering period HT tolerance. In addition, cultivars possessing late season field drought tolerance (stay green traits) appeared to be HT tolerant, suggesting a possible relationship between drought and HT responses [128]. An interesting area of research yet to be explored is the cytoplasmic effects on HT in sorghum. Sorghum has about five different cytoplasmic male sterility (CMS) lines; A1 CMS are largely exploited worldwide, and other CMS lines’ potential uses are being evaluated for their commercial hybrid breeding (Govindaraj Pers. Commn.). Since HT effects are attributed to spatial and temporal nature, variations bring significant masking epistasis effects while studying the traits genetics, the heritability of HT tolerance traits. In general, low to moderate heritability like in sorghum or other similar crops suggests the feasibility of genetic enhancement, as open-pollinated varieties or broad-based hybrids (top-cross and three-way cross) which depends on traits gene effects. Two lines, B35 and BTx3197, were used as HT tolerant sources in sorghum improvement programs [128,129]. HT and drought tolerance are independent traits, however, discriminating the confounding effects during breeding pipeline evaluation and their selection is challenging. This confounding effect also assumes that selection for drought tolerance traditionally improves HT tolerance in sorghum. The proposed breeding scheme (Figure 5) is largely in the early stages of understanding the crop variability spectrum to HT and can be improved with future understating of associated traits and their efficient screening tools [130].

At ICRISAT, about 1000 breeding lines (600 B-lines, 300 R-lines and 100 varieties) are screened for flowering and seed set. Of the 1000 lines, the flowering time of only eight lines (ICSR 14001, ICSR 8, ICSR 21, ICSB 55, ICSB 84, ICSB 603, ICSV 162, ICSV 376) is unchanged in the rainy and post-rainy season with 100% seed set, indicating the potential HT tolerance of these lines [131]. It is important to note that sorghum photo-insensitivity may be linked to HT expression and this needs further investigations. In India, it is critical to plant the field screening material in the first week of March (southern and central zone) where the maximum temperature noncoinciding during sorghum flowering in April‒May will give appropriate data on HT tolerance traits including flowering time, seed set %, and panicle harvest index as proxy traits for selecting for HT tolerance in sorghum.

As described in drought tolerance (stay green) breeding, HT tolerance breeding requires systematic relevance traits discovery more than pollen germination and its viability to achieve a higher rate seed set under HT. The primary (contributing directly to HT tolerance) and secondary traits (indirectly contributing HT tolerance) can play a key role in the proposed breeding scheme and selection strategies. The integrated breeding of phonemics and genomics may provide a new opportunity for incorporation and validation of the reported 26 QTLs for five traits (Figure 4) claimed for HT tolerance in breeding pipelines, which may yield a wider spectrum of HT tolerance progenies with better agronomic traits. The maximum QTLs identified for leaf firing as a HT tolerance trait may attract accelerated validation in different genetic backgrounds to assess the co-segregation pattern of multiple QTLs to pyramid HT tolerance traits in breeding pipelines. HT tolerance breeding pipelines are likely to be smaller compared to mainstream breeding pipelines. They are appropriate to be handled independently at early breeding stages to understand the target level of HT (average higher temperature prevalence in target growing crop ecology) and the type of genetic materials suitable for target ecology and amenable for trait introgression. A simulation study was conducted using a sorghum model to quantify the potential benefits of altering the crop cycle, enhancing yield potential traits, and incorporating drought and HT tolerance in India and Mali [132]. There were benefits in grain yield observed for drought and HT tolerance in different locations. Overall, they concluded that different combinations of traits would be needed to increase and sustain sorghum yield in current and future climates [132].

## 13. Conclusions

Drought and HT stress causes significant and negative impacts on various physiological, genetic, and molecular changes that adversely affect sorghum growth, development, yield, and yield components. The resilience of future sorghum varieties and hybrids to climate change can be improved with better understating of physiological and molecular basis of tolerance or susceptibility. Mechanisms of tolerance or susceptibility to drought or HT stress indicate that the mechanism of abiotic stress tolerance has been relatively less explored in sorghum compared with rice or wheat. Systematic research needs to be conducted on the identification of primary and secondary traits associated with tolerance at above critical stress levels. The severity of drought and HT stress and negative impacts on growth and yield will be greater if these stresses occur at the reproductive stages of crop development which are more sensitive to stresses. The primary effects of drought stress include lower tissue water content leading to decreased membrane stability, loss of green leaf area, carbon assimilation, and partitioning leading to lower growth, biomass, and yields. Similarly, the membranes are the primary sites of action for temperature extremes. High temperature stress leads to lower membrane stability, lower carbon assimilation, greater respiration, and decreased floret fertility, leading to lower seed numbers, seed size, and yield. The key traits associated with drought tolerance include stay green, canopy temperature depression, limited transpiration, higher reproductive success, and root architecture, while the key traits associated with HT stress include higher membrane stability, greater gamete viability and reproductive success leading to higher seed number, canopy temperature depression, favorable respiration, and early flowering. Many QTLs, genes, and molecular mechanisms associated tolerance or susceptibility for some of traits are known, but they are not clearly understood and must be systematically studied and used in the breeding programs. Further studying the relationship between traits related to drought and HT is essential to devise a combined selection strategy as these two stresses commonly occur in many sorghum-producing geographies. The recent developments in genomics and precision genotyping and phenotyping techniques will pave the way to identify the potential genes underlying drought and HT traits. High-throughput phenotyping methods (e.g., leasyscan and use of imaging techniques) to characterize the large and diverse breeding material and populations are needed. High-throughput precision phenotyping of key traits of tolerance needs further exploration to improve the abiotic stress tolerance of sorghum. Crosstalk among crop breeding and allied disciplines (e.g., genetics, statistics, physiology, genomics, and agronomy) while designing the drought and HT tolerant breeding approach and pipelines is critical to accelerate drought and HT resilient product development. A holistic contribution-based collaborative breeding team and the pipeline must be employed to improve stress tolerance and increase genetic gains in sorghum, particularly in semi-arid regions of SSA and SA where sorghum is a key crop for food and nutritional security with multiple uses (food, feed, and fuel).

## Figures and Tables

**Table 1:** List of genes and their function reported under drought and/or high temperature stress in sorghum.

S. No.	Gene(s)	Function	Reference
1.	*SbSNAC1*	Confers drought tolerance	[68,90]
2.	*SbNAC052*, *SbNAC073* and *SbNAC116*	Negatively regulate the expression of stress responsive genes	[75]
3.	*SbNAC014*, *SbNAC035* and *SbNAC041*	Transcriptional activator, plays important role during post-flowering drought stress in sorghum	
4.	*SbNAC037*	Nutrient remobilization during drought stress	
5.	*SbDof12, SbDof19* and *SbDof24*	Imparts tolerance during the onset of drought stress	[73]
6.	*SbDof21*, *SbDof22*, *SbDof23*, *SbDof25*, *SbDof27* and *SbDof28*	Imparts tolerance during later stage of drought stress	
7.	*SbGH3* and *SbLBD*	Involved in the auxin and drought stress signaling cross talk	[74]
8.	*SbPIN4/5/8/9/11*	Exhibits ABA-induced expression	[79]
9.	*SbWRKY30*	Positive regulator and highly expressed in sorghum taproot and leaves	[77]
10.	*SbWRKY74*, *SbWRKY75*, *SbWRKY19*, *SbWRKY5*, *SbWRKY45*, *SbWRKY79*, *SbWRKY25*, *SbWRKY 83*, *SbWRKY 16* and *SbWRKY72*	Involved in the drought stress response during seedling, flowering, and dough stages	[78]
11.	*SbWRKY46*	Implicated in drought stress tolerance	[85]
12.	*SbP5CS1* and *SbP5CS2*	Highly expressed under drought stress. *SbP5CS1* shows high expression in vegetative and reproductive organs, whereas *SbP5CS2* shows expression in all the tissues	[84]
13.	*SbAO3*, *SbASPG1*, *SbCIPK15*, *SbCKX4*, *SbGST*, *SbGsSRK*, *SbMAPKKK7*, *SbMAPK10*, *SbER2-1*, *SbZFP*, *SbLAH2*, *SbEXOB1*, *SbPUB43*, *SbPPR3* and *SbTLP1b*	Implicated in drought stress tolerance	
14.	*SbER2-1*	Important role in drought stress responses and implicated in photosynthetic systems and phenylpropanoid metabolism in crop plants.	[96]

**Table 2:** Germplasm identified for drought stress tolerance at the International Crops Research Institute for the Semi-Arid Tropics (ICRISAT), India.

Sl. No	Genotype	Group	Centre	Pedigree/Parent Sources	Reference
Emergence					
1.	IS 4405	Germplasm	ICRISAT	IS 4405	[121]
2.	IS 4463	Germplasm	ICRISAT	IS 4463	
3.	IS 17595	Germplasm	ICRISAT	IS 17595	
4.	IS 1037	Germplasm	ICRISAT	IS 1037	
5.	VZM1-B	Inbred	NA *	Unknown	
6.	2077 B	Germplasm	ICRISAT	IS 18790	
7.	IS 2877	Germplasm	ICRISAT	IS 2877	
8.	IS 1045	Germplasm	ICRISAT	IS 1045	
9.	D 38061	Inbred	NA	Unknown	
10.	D 38093	Inbred	NA	Unknown	
11.	D 38060	Inbred	NA	Unknown	
12.	ICSV 88050	Variety	ICRISAT	(IS 27043 × IS 10469)-1-1-BK-1-BK-BK	
13.	ICSV 88065	Variety	ICRISAT	(IS 24737 × IS 18729)-2-1-BK-1-BK-BK	
14.	SPV 354	Variety	NRCS	Unknown	
Seedling					
15.	ICSB 3	Inbred	ICRISAT	[(BTx 622 × UChV2)B lines bulk]-4-2-1-1	[121]
16.	ICSB 6	Inbred	ICRISAT	[(BTx 623 × UChV2)B lines bulk]-3-1-4-3	
17.	ICSB 11	Inbred	ICRISAT	[(BTx 624 × UChV2)B lines bulk]-5-1-1-1	
18.	ICSB 37	Inbred	ICRISAT	[(BTx 623 × MR 862)B lines bulk]-5-1-2-5	
19	ICSB 54	Inbred	ICRISAT	Diallal 346-8556-2-1	
20.	ICSB 88001	Inbred	ICRISAT	[(ICSB 22 × ICSB 53) × Diallel 7-2-862]-1-1	
Pre-flowering					
21	DKV 1	Variety	NA	Unknown	[121]
22.	DKV 3	Variety	NA	Unknown	
23.	DKV 7	Variety	NA	Unknown	
24.	DJ 1195	Variety	NA	Unknown	
25.	ICSV 272	Variety	ICRISAT	[(M35-1 × M-1009)-3-2-1 × F5-6]-5-2-3-1-1	
26.	ICSV 273	Variety	ICRISAT	[(M35-1 × M-1009)-3-2-1 × F5-6]-5-2-3-1-2	
27.	ICSV 295	Variety	ICRISAT	[(M-35-1 × M-1009)-3-2-1 × 6 F5’S]-5-1-4-1-1	
28.	ICSV 378	Variety	ICRISAT	[CSV4 (M 35-1 × M 1007)-3-1-1]-1-1-1-1-1	
29.	ICSV 572	Variety	ICRISAT	(D71283 × 2219 B)-1-1-1-2-2	
30.	ICSB 58	Inbred	ICRISAT	(2219B × 148)-8-1-1-1-2	
31.	ICSB 196	Inbred	ICRISAT	Unknown	
Post-flowering					
32.	IS 19153	Germplasm	ICRISAT	IS 19153	[122]
33.	IS 23514	Germplasm	ICRISAT	IS 23514	
34.	IS 29392	Germplasm	ICRISAT	IS 29392	
35.	RS 585	Inbred	NRCS	(CS 3541 × M 35-1) × Nandyal Rabi Local	
Terminal drought					
36.	E 36-1	Variety	ICRISAT	IS 30469	[121]
37.	DJ 1195	Variety	NA	Unknown	
38.	DKV 3	Variety	NA	Unknown	
39.	DKV 4	Variety	NA	Unknown	
40.	DKV 17	Variety	NA	Unknown	
41.	DKV 18	Variety	NA	Unknown	
42.	ICSB 17	Inbred	ICRISAT	[(BTx 623 × 1807B)B lines bulk]-18-1-1	
Maturity					
43.	CSH 1	Hybrid	NRCS	CK60A × IS 84	[123]
44.	CSH 6	Hybrid	NRCS	2219A × IS 3541	
45.	NK 300	Variety	NA	Unknown	
46.	M 35-1	Variety	ARS, Mohol, Maharashtra	IS 37185	
47.	SPV 86 (CSV 8R)	Variety	NRCS	CS 3541 × Tall Mutant	

* NA—not available.

**Table 3:** QTLs identified for drought tolerance traits in sorghum.

Sl. No	Type of Population	Cross	Trait(s)	Number of QTLs	Name of QTLs/Markers	Reference
1.	NILs	RTx7000	Stay green	4	*Stg1*, *Stg2*, *Stg3*, *Stg4*	[17]
2.	Inbred panel	NA *	Drought tolerance	3	*Xtxp69*, *SbAGA01*, *SbAGB03*	[85]
3.	RILs	Tx7078 × B35	Pre-flowering drought	6	*b465/140*, *tK12/115*, *bDll/65*, *tM5/75*, *tC13/150*, *bC18/820*	[113]
4.	RILs	SC56 × Tx7000	Pre-flowering drought	3	*Stg A*, *Stg G*, *Stg J*	[112]
5.	RILs	TX7000	Stay green	3	*Stg1*, *Stg3*, *Stg4*	[114]
6.	RILs	TX7078 × B35	Stay green	2	*AE80%-E1–1*, *AE80%-E1–2*, *AE80%E1–3*,	[115]
7.	RILs	M35-1 × B35	Stay green	3	*Stg2*, *Stg3*, and *StgB*	[118]
8.	RILs	RSG04008-6 × J2614-11	Stay green associated trait	5	*Gl 7*, *Gl 14*, *Gl 21*, *Gl 28*,	[119]

* NA–Not available.

## Data Availability

The study did not report any data.
